# Effective Method Using a Stool in Cardiopulmonary Resuscitation (CPR) on Dialysis Chair

**DOI:** 10.1155/2020/5691607

**Published:** 2020-07-27

**Authors:** Takeshi Ifuku, Takashi Hitosugi, Yoshfumi Kawakubo, Tomoyuki Tanaka, Kazuto Doi, Takeshi Yokoyama

**Affiliations:** ^1^Department of Cardiology, Haraguchi Hospital, 6-11-15 kotabe Sawara‐ku, Fukuoka City, Fukuoka 814-0032, Japan; ^2^Department of Dental Anesthesiology, Faculty of Dental Science, Kyushu University, 3-1-1 Maidashi, Higashi-ku, Fukuoka 812-8582, Japan; ^3^Osaka Jikei College Office for Establishment of University, 1-2-8, Miyahara, Yodogawa-ku, Osaka 532-0003, Japan; ^4^Osaka College of High Technology, Department of Clinical Engineering, 1-2-43, Miyahara, Yodogawa-ku, Osaka 532-0003, Japan; ^5^Department of Medical Care and Welfare Engineering, School of Industrial and Welfare Engineering, Tokai University, 9-1-1 Toroku, Higashi-ku, Kumamoto-shi, Kumamoto 862-8652, Japan

## Abstract

**Background:**

Heart failure is the leading cause of death in dialysis patients. Cardiac arrest due to hypotension may also occur during dialysis therapy. If cardiac arrest is elicited, manual chest compressions (MCCs) should be started as soon as possible. However, all types of dialysis chairs are not stable for MCC, because there is no steady support between the backboard of the dialysis chair and the floor. These conditions may alter the effectiveness of MCC.

**Methods:**

We investigated whether a round chair is effective in supporting the dialysis chair for MCC. Four adult males performed MCC on a mannequin placed on three dialysis chairs. MCC was performed in sets of 2 (each set was 100 times per minute) per person, with and without a round chair. A total of 4,800 compressions were performed by four executors.

**Results:**

When the chair was not used as a stabilizer, the mean values of the fluctuation range were 20.8 ± 8.1 mm, 18.7 ± 5.5 mm, and 12.8 ± 1.8 mm, respectively. When the chair was used, the mean values of the fluctuation range were 6.1 ± 1.1 mm, 7.5 ± 2.1 mm, and 1.0 ± 0 mm, decreasing by 70%, 59%, and 92%.

**Conclusion:**

MCC performed with the stool under the backrest as a stabilizer was effective in supporting the dialysis chair.

## 1. Background

The number of dialysis patients has increased almost every year, being more than 330,000 in Japan. Japan, with 2,599 patients per million general population, has the second largest prevalence of treated end-stage renal disease [1]. Heart failure is the leading cause of death. When combined with 3.8% myocardial infarction, the ratio is 27.8% of the total. It is reported that heart failure is also the second leading cause of death within the year of introduction of dialysis therapy for patients in 2017, accounting for 20.8% [2]. There are also reports of approximately 5% sudden cardiac death [3]. In hemodialysis therapy, treatment is generally performed for 4 to 5 hours on a dialysis bed or medical chair (hereinafter, referred to as “dialysis chair”).

Dialysis patients are at risk of developing hypotension and cardiac arrest. In cardiopulmonary resuscitation (CPR), manual chest compressions (MCC) and defibrillation must be started as soon as possible. The 2015 American Heart Association (AHA) Guidelines emphasize the importance of pushing hard and fast and of minimizing interruptions during compression. In addition, it is recommended that chest compressions in adults be performed at a depth of 5 cm or more and 6 cm or less, from 100 to 120 times per minute [4]. Therefore, to maximize the effectiveness of MCC, the patient should be placed supine on a firm, stable plane [2]. Patients on dialysis beds can be treated by placing a resuscitation board just under the chest. However, when a dialysis chair is used for treatment, even if the backrest is tilted horizontally to the floor and the resuscitation board is placed, there will be no stable support between the backrest of the dialysis chair and the floor. Therefore, it is presumed that the backrest shakes and becomes unstable when MCC is applied. Although there is a report [5] verifying the usefulness of MCC on a dental chair and a report [6] that MCC can be effectively performed, there is no report of MCC performed on dialysis chairs.

The objective of this study was to evaluate the effectiveness of using a round chair as a stabilizer between the backrest and the floor in different types of dialysis chairs.

## 2. Materials and Methods

### 2.1. Materials

Three different types of dialysis chairs, Model 1 (CM2-020-I®; TACHI-S&P.LTD, Tokyo, Japan), Model 2 (Ipsia Tre®; Okamura, Co., Kanagawa, Japan), and Model 3 (SD-5500; Okamura, Co., Kanagawa, Japan), and a mannequin for CPR training (Ambu® man model C torso, Ambu A/S, Ballerup, Denmark) were used in the study.

### 2.2. MCC Executors

MCC was performed by four healthcare providers who completed AHA-certified basic life support course in this study: executor 1: 46-year-old man, 171 cm, 60 kg; executor 2: 27-year-old man, 169 cm, 80 kg; executor 3: 21-year-old man, 175 cm, 67 kg; executor 4: 41-year-old man, 168 cm, 62 kg.

### 2.3. Mannequin Installation Method and Measurement of the Vertical Displacements of the Backrest

**Figure 1:**
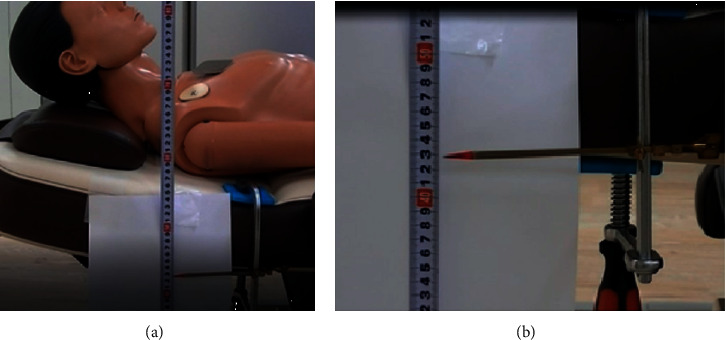
(a) The placement of mannequin and guides for measuring the vertical displacements of the backrest. (b) An enlarged view of the pointer attached to the backrest.

A mannequin for CPR was placed on a dialysis chair, which was tilted horizontally. The head was placed on the headrest of the dialysis chair and the chest of the CPR mannequin was placed horizontally using a level device (Z-340; Hozan Co., Osaka, Japan) (Figure 1(a)). To measure the vertical displacements of the backrest, a metal pointer was attached horizontally to the backrest of the dialysis chair, and the position was just below the MCC implemented portion (Figure 1(b)).

### 2.4. Comparison of MCC Implementation Methods and Measurements

**Figure 2:**
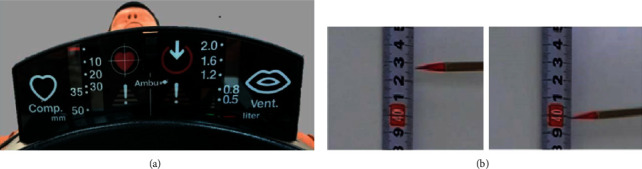
Compression depth indicator on the mannequin (a) and pointer taken with a camcorder (b).

For each dialysis chair, MCC was performed in sets of 2 (each set was 100 times per minute) per person, with and without a round chair. A total of 4,800 compressions were performed by four executors. The compressions were located in the middle of the chest and in the lower half of the sternum according to the European Resuscitation Council Guidelines for Resuscitation (2015) and the 2015 AHA Guidelines. The speed was adjusted to the sound by setting the metronome at 100 times/minute. The depth of MCC was always between 3.5 cm and 5 cm. The indicator attached to the mannequin (Figure 2(a)) was green at compression depths of 3.5 cm to 5 cm and red at compression depths of less than 3.5 cm and more than 5 cm. When performing MCC, the executors confirmed that the depth of the compression reached the specified value and made sure to perform with compression assistance. The vertical displacements of the backrest were captured with a camcorder (GZ-E180; JVCKENWOOD Co., Kanagawa, Japan), and the video data were transferred to a computer (Model 1631; Microsoft Co., WA, USA). Using the scale of the measure shown in Figure 2(b) as a reference, we measured the difference in the position of the indicator between when the pressure was applied and released to measure the width of movement of the backrest (Figure 2(b)). Compressions that did not reach the specified value were excluded.

### 2.5. Chair Arrangement to Stabilize the Dialysis Chair

**Figure 3:**
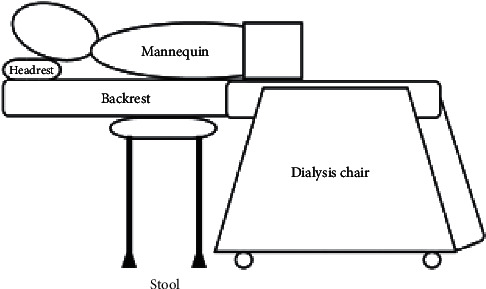
The layout of a dialysis chair and a stool.

In order to verify the stability when a stool was placed between the dialysis chair and the floor as a support, the backrest and the chair were placed horizontally in close contact (Figure 3). The chair was placed just below the MCC implemented area of the mannequin.

### 2.6. Statistical Analysis

For each dialysis chair, the vertical displacements of the backrest were compared between with and without the use of a stool. In addition, comparisons for all enforcements and among executors were performed. The normality of each data group was tested with the Shapiro–Wilk test (with the function shapiro.test) for the vertical displacements of the backrest of the dialysis chair during MCC (*p* < 0.05). The Kruskal–Wallis test was used to obtain differences between the data groups, considering that the data groups were not normally distributed. The Steel–Dwass test was used as a post hoc test (*p* < 0.05). For statistical analysis, programming language R (version 3.4. 3; The Comprehensive R Archive Network, USA) and Excel statistics 2012 for Windows (R) (Social Information Service Co., Ltd.) were used.

## 3. Results

The vertical displacements of the backrest during MCC were measured 4,800 times, with and without a round chair. 17 out of 4,800 were excluded because they did not meet the criteria, and a total of 4,783 trials were compared.  Table 1 shows the vertical displacements with a round chair (hereinafter, referred to as “fix”) or without a round chair (hereinafter, referred to as “free”) for Model 1. The vertical displacements of the backrest of “fix” was lower than the vertical displacements of “free” among all executors (*p* < 0.01). In the compressions performed by executor 4, the mean value of the vertical displacements of the backrest decreased by 77.7% in “fix” compared with “free.” Table 2 shows the vertical displacements of Model 2 without and with a stool. The vertical displacements of the backrest of “fix” were lower than the vertical displacements of “free” among all executors (*p* < 0.01).  Table 3 shows the vertical displacements of Model 3. Particularly, the vertical displacements of “fix” were significantly lower than the fluctuation range of “free” among all executors (*p* < 0.01).  Table 4 shows the vertical displacements of the backrest without and with a stool for all dialysis chairs. The vertical displacements of “fix” were significantly decreased compared with “free,” among all dialysis chairs (*p* < 0.01). The decrease rate was 70.9% for Model 1, 59.8% for Model 2, and 92.2% for Model 3, indicating improved stability for all dialysis chairs. However, the vertical displacements were different for each dialysis chair regardless of whether there was the stool or not. For Model 3, the vertical displacements of the backrest were 12.8 ± 1.8 mm in “free” and 1.0 ± 0 mm in “fix.” The mean value of the vertical displacements in “fix” decreased by 92% compared with the case in “free.” In the comparison of MCC performed by the executors, the vertical displacements of the backrest with the stool were significantly lower than those without one, and the stability during MCC was improved among all the executors.

## 4. Discussion

During dialysis, one of the most common complications is cardiopulmonary arrest. Most common complication of dialysis is cardiopulmonary arrest. It was caused during dialysis treatment 70%, postdialysis 21%, and predialysis 10% [7]. In addition, according to a statistical survey by the Japanese Society for Dialysis Therapy, the rate of cardiopulmonary arrest during dialysis treatment was not reported, but heart failure was the leading cause of death, accounting for 27.2% [8].

MCC requires the patient to lie in the supine position on a firm, stable plane and the rescuer to stand beside the patient's chest and initiate compressions as soon as possible. However, during dialysis treatment, it is not easy to carry the patient from the dialysis chair to the floor because the patient's devices are connected with a blood circuit and the length of the circuit may not be sufficient. Therefore, MCC is performed on the dialysis bed or chair. If the patient being treated on the dialysis bed has a cardiopulmonary arrest, a certain level of stable MCC can be performed by placing a resuscitation board on the bed. However, when treatment is performed on a dialysis chair, there will be no support between the backrest of the dialysis chair and the floor, and hence, a large vertical displacement occurs when the patient's chest is compressed, making it difficult to perform stable MCC. Also, in the case of MCC performed on a dental chair, similar to a dialysis chair, there is no support between the backrest and the floor, and therefore, when MCC is applied, the backrest shakes and becomes unstable. In order to improve the situation, the effectiveness of using a round chair as a support directly below the MCC implemented area between the backrest of the dental chair and the floor has been reported [4], and this method is recommended in the ERC Guideline 2015 [9].

In this study, using the same method as in the report [4], we verified three types of dialysis chairs, with and without a round chair as a support. As a result, the vertical displacements when using a round chair was significantly reduced among all dialysis chairs compared to those when the chair was not used. In Model 1 and Model 2, the vertical displacements decreased significantly but the vertical displacements varied. In Model 3, there was no variation in the vertical displacements among all the executors. It could be said that placing a stool under the backrest as a stabilizer and performing MCC is effective regardless of the model. Hence, it is useful to use a stool as a support for MCC on a dialysis chair with a reclining function.

However, dialysis chairs are often used in a sitting position and can be operated by patients themselves. The dental chairs are intended for treatment in a supine position and not to be operated by the patients themselves. In addition, during a dialysis treatment, the patient will spend a typical treatment time of 4 hours on the dialysis chair. Therefore, the chairs are made in consideration of comfort and relaxation, and many of them have backrest with high cushioning property. Both types of chairs are similar, but they differ in some details such as usage, thickness, hardness, and structure. For chairs with high cushioning properties, it is necessary to consider the use of a stool as a stabilizer.

## 5. Conclusions

MCC performed with the stool under the backrest as a stabilizer was effective in supporting dialysis chairs during treatment.

## Figures and Tables

**Table 1:** The displacements without and with a stool for Model 1.

Model 1	Free		Fix		*p* value	Decrease rate (%)
	Mean	SD	Mean	SD		
Executor 1	12.9	0.9	6.3	0.6	<0.01	51.0
Executor 2	20.1	9.1	5.2	1.1	<0.01	74.3
Executor 3	21.7	6.9	6.4	1.3	<0.01	70.6
Executor 4	28.7	1.9	6.4	0.6	<0.01	77.7

Free: without a stool; fix: with a stool.

**Table 2:** The displacements without and with a stool for Model 2.

Model 2	Free		Fix		*p* value	Decrease rate (%)
	Mean	SD	Mean	SD		
Executor 1	16.9	1.5	4.6	1.4	<0.01	73.0
Executor 2	18.3	3.1	7.0	0	<0.01	61.7
Executor 3	18.3	5.7	8.5	0.5	<0.01	53.4
Executor 4	21.5	8.2	10.1	0.3	<0.01	53.2

Free: without a stool; fix: with a stool.

**Table 3:** The displacements without and with a stool for Model 3.

Model 3	Free		Fix		*p* value	Decrease rate (%)
	Mean	SD	Mean	SD		
Executor 1	15.0	0.9	1.0	0	<0.01	93.3
Executor 2	11.3	1.6	1.0	0	<0.01	91.2
Executor 3	12.3	0.9	1.0	0	<0.01	91.9
Executor 4	12.6	1.4	1.0	0	<0.01	92.1

Free: without a stool; fix: with a stool.

**Table 4:** The vertical displacements of the backrest with and without a stool for all dialysis chairs.

	Free		Fix		*p* value	Decrease rate (%)
	Mean	SD	Mean	SD		
Model 1	20.8	8.1	6.1	1.1	<0.01	70.9
Model 2	18.7	5.5	7.5	2.2	<0.01	59.8
Model 3	12.8	1.8	1.0	0	<0.01	92.2

Free: without a stool; fix: with a stool.

## Data Availability

The data used to support the findings of this study are available from the corresponding author upon request.
